# Account of Medical Education in Edinburgh, with Remarks

**Published:** 1801-10

**Authors:** 


### *Account of Medical Education in Edinburgh, with Remarks.*

---

To the Editors of the MEDICAL and PHYSICAL JOURNAL.

Gentlemen,

AS many of your readers may feel a satisfaction in being acquainted with the manner in which medical education is conducted, in a seminary so much celebrated as Edinburgh, I take the liberty of sending you, for insertion in your respectable Publication,

Publication, ſome details on that ſubject, which, as I ſtudied and graduated at that Univerſity, I am able to give you with tolerable accuracy.

It may be proper to premiſe a few general remarks on the origin and conſtitution of this ſeminary.

The Univerſity of Edinburgh was founded by James the VIth of Scotland, about the year 1581, and is denominated in all official writings, *Academia Jacobi Sexti, Regis Scotorum quæ Edinburgi est.* It conſiſts of a Principal and Profeſſors, who have ſmall ſalaries, and all of them, except the Regius Profeſſors, are appointed by the Town-council of Edinburgh, who have likewiſe the power of inſtituting new Profeſſorſhips when they ſhall ſee occaſion. The Principal, with the whole body of Profeſſors, form the Senatus Academicus, which enacts laws for the regulation of the College, and ſettles all matters relating thereto. Degrees are conferred, at certain periods, by the Principal, with the conſent and approbation of this body. There is an endowment of a few pounds per annum for a ſmall and limited number of Burſars, but there are no Fellows.

Moſt of the Profeſſors, and the whole number of ſtudents, live in the town; the latter wear no particular dreſs, are ſubject to no College laws, and may attend the lectures at ſuch times only as they pleaſe. There is indeed a ſmall fine for abſence or inattention, at one or two of the junior claſſes, as the Latin and Greek. The College exerciſes, beſides thoſe of conſtruing, tranſlating, or demonſtrating, at the Latin, Greek, and Mathematical claſſes, are writing occaſional eſſays on ſubjects connected with their ſtudies; at the Logic, the higher Latin and Greek claſſes, the Divinity, and of late years at the Moral Philoſophy. The only claſs, I believe, at which there is any examination on what has been previouſly heard, is the Logic. It is rather to be lamented that there are not more frequent opportunities of competition in the various claſſes of this Univerſity; and it is a circumſtance which muſt naturally excite conſiderable wonder among thoſe who are informed of it, that there is not a ſingle medal or prize of any kind ever offered for the beſt eſſay on any ſubject, whether literary or philoſophical. When it is conſidered how great is the inducement afforded to induſtry by an honorary reward of this kind, how trifling is the expence attending it, and how very many there are of diſtinguiſhed rank, fortune and reputation, who have had the elements, if not the whole of their education, at this Univerſity, it is extremely ſingular that no endowment of this purpoſe has ever been made. The Profeſſors themſelves, who amount to a conſiderable number, and are highly reſpectable for their learning and talents, might, by a very ſmall individual contribution, raise a sufficient sum annually for defraying the expence of a small number of prize medals.

The Professorships in the University of Edinburgh are divided into four faculties, viz. the *Faculty of Theology*, of *Law*, of *Medicine*, and of *Arts*; and the whole number of Professors amounts to about twenty-four.

In the *Faculty of Theology* is comprized Divinity, Church History, Hebrew and Oriental Languages.

In the *Faculty of Law*, Civil Law, Law of Nature and Nations, Universal History, and Scot's Law.

In the *Faculty of Medicine*, Anatomy and Surgery, Practice of Physic, Chemistry, Materia Medica, Theory of Physic, Botany, and Midwifery.

In the *Faculty of Arts*, Latin or Humanity (literæ humaniores), Greek, Mathematics, Logic, Moral Philosophy, Natural Philosophy, Rhetoric and Belles Lettres, Natural History, Practical Astronomy, Agriculture.

Till near the revolution there were no medical Professors at this University, and even till the year 1720 they were merely titular. About that period the late celebrated Professor Monro commenced his anatomical lectures, and from that we may date the formation of the Medical School in Edinburgh, which has gradually arisen to a degree of celebrity unequalled by any other in Europe.

From the above enumeration, it appears that the medical Professorships are seven in number. That of Midwifery is not however on the same footing with the others, as the Professor has no share in the medical examinations, nor is it necessary for a student to attend this class before he is eligible to a medical degree.

The Lectures commence in the latter end of October, with the exception of Midwifery, of which there are three courses in the year, viz. two in the winter and one in the summer, and last until the latter end of April or the beginning of May. The Botanical Lectures are only given in the summer season, during the months of May, June, and July, at the Botanical Garden, a short distance from Edinburgh. The present Professor of Chemistry has for some time given a summer course of lectures which lasts three months.

The first few lectures in the season are always occupied with the history of the science, by way of introduction; and as the sacraments of the church of Scotland, which occur twice a year, give an interruption to them for a week in the beginning of November, the particular business of the course is not entered upon till about the 10th or 11th of that month. The lectures are an hour each in length, except the anatomical, which which is an hour and half at leaſt. They are given on every week day except Saturday, and on that day ſome of the Profeſſors have occaſion to lecture during a part of the ſeaſon, in order to get through the buſineſs of the courſe.

The following are the names of the preſent Profeſſors, and the hours at which they lecture:

| | | | |
|---|---|---|---|
| Dr. Monro<br>Dr. Monro, jun. | Anatomy - - - - - - | | 1 P. M. |
| Dr. Gregory - - | Practice of Medicine - - | | 9 A. M. |
| Dr. Hope - - - | Chemiſtry - - - - - - - | | 10 A. M. |
| Dr. Duncan - - | Theory or Inſtitutes of Med. | | 11 A. M. |
| Dr. Home<br>Dr. Home, jun. | Materia Medica - - - | | 8 A. M. |
| Dr. Rutherford - | Botany - - - - - - - - | | 8 A. M. |
| Dr. Hamilton<br>Dr. Hamilton, jun. | Midwifery - | in Winter<br>in Summer | 3 P. M.<br>10 A. M. |

Any perſon who fees a claſs may attend the lectures; it is neceſſary, however, to be afterwards enrolled in the College books at the matriculation, to be able to procure any official document of academical education, and to have acceſs to the valuable collection of books of which the college library conſiſts. This noble library is enriched with a copy of every book which is entered at Stationers' Hall, and is aided by a ſmall ſum paid by each ſtudent at his matriculation. The books may be procured, to a limited number, by ſtudents, on their depoſiting the value with the librarian, who returns it on receiving the book.

The whole number of ſtudents at the Univerſity is from 1000 to 1300, of whom between four and five hundred are medical. The Profeſſor of Anatomy has never fewer than three hundred attending him, and ſometimes many more. The other claſſes, particularly thoſe of the Practice of Medicine and Chemiſtry, are alſo well attended.

To the Profeſſor of Anatomy belongs a very capital collection of preparations, which may be inſpected by the ſtudents in the lecture room, or in ſome cloſets adjoining it, in the courſe of the ſeaſon. He is always able to provide a ſufficient number of ſubjects for public demonſtration, but there are no opportunities of private diſſection afforded in the College.

Students who attend the Midwifery claſs have the advantage of practice at the Lying-in Hoſpital. Connected with the medical education of the College is the attendance at the Royal Infirmary, which is a building capable of containing above two hundred patients, and ſupported principally by voluntary contribution. The medical and ſurgical patients are kept in different different wards, and from the medical are felected about fifteen males, and as many females, with the moft interefting complaints, who are put into feparate wards, denominated *Clinical*, and are attended by a medical Profeffor of the College, of whom there are generally four who take the duty alternately. The profeffional bufinefs of the Infirmary is performed by two ordinary phyficians, appointed for a permanence, with falaries; two furgeons,* a clinical phyfician, two phyficians' clerks, a houfe furgeon, and two clinical phyficians' clerks. The phyficians' clerks and houfe furgeon are appointed by the managers, and refide in the hofpital; the clinical phyficians' clerks are appointed by the clinical phyfician for the time of his attendance, and do not refide. The patients are feen by the phyfician or furgeon having the care of them, every day, except Sunday, at twelwe o'clock.

The manner in which the bufinefs of the Hofpital is conducted, reflects much credit on all concerned in it, and is extremely conducive to the improvement of the ftudent. On the admiffion of any patient, whether furgical or medical, the houfe furgeon, or phyficians' clerks, take down an accurate account of the fymptoms, and hiftory of the complaint, in a book for the purpofe, in which are alfo inferted the daily progrefs and treatment, dictated by the attending furgeon or phyfician. Thefe books are, at certain times of the day, left in a particular room for the infpection of the ftudent; and as they are liable to feverity of criticifm, both as to ftyle and accuracy, it is the intereft of all parties that they fhould be correct. The furgical cafes of the Hofpital form the bafis of a valuable courfe of Lectures, given twice a week during the winter months, by Mr. Ruffell.

Patients admitted into the clinical wards are the peculiar objects of attention. An accurate hiftory of their complaints is taken by one of the clerks on their admiffion, and a very full report of the ftate of the patient, with the progrefs and treatment of the difeafe, is every day dictated by the clinical Phy-

---

* The care of the furgical patients of the Hofpital formerly belonged to the whole College of Surgeons, who took the duty by rotation, for two months at a time; the fucceffor acting as an affiftant until it became his turn to officiate. Dr. Gregory, while Prefident of the College of Phyficians of Edinburgh, publifhed an addrefs to the managers on the fubject of the furgical attendance at the Infirmary, and fucceeded fo far as to induce them to make material alterations in it. There are now, I believe, on the eftablifhment, fix Hofpital Surgeons, who are appointed for a permanence, with fix affiftant furgeons under them. Two of thefe furgeons act for two years at a time, and divide the patients. There is alfo a certain number of dreffers appointed by the furgeons.

fician, who alfo gives Lectures on thefe cafes at the College every Tuefday and Friday, at five o'clock. There are two clinical Phyficians in winter and one in fummer, each of them officiating in this fituation three months.

The fee for the clinical, and each courfe of medical lectures, at the College, is three guineas, with a fmall fum to the janitor and fervants. Every part of the Hofpital, whether medical, furgical, or clinical, is open to the ftudent for three guineas annually; and this fee, as well as that for the clinical lectures, is, I believe, leffened after a certain attendance. The ftudents are at liberty to vifit any part of the Hofpital, at any time of the day, to make their perfonal obfervations on the ftate of the patients, and compare the reports dictated by the phyficians or furgeons with the appearances of the difeafe.

Before any gentleman can be examined for a medical degree in Edinburgh, he muft bring forward documents of having ftudied medicine three years, at that or fome other Univerfity. The teftimonial of that part of his education which he received in Edinburgh, is given him from the records of the Univerfity by the Secretary. As it may be acceptable to fome of your readers to be informed of the regulations with regard to medical degrees in Edinburgh, I fhall prefent them with nearly a literal tranflation of the ftatutes on this fubject, known by the name of *Statuta Solemnia.*

STATUTES of the UNIVERSITY of EDINBURGH on the fubject of Medical Degrees; propofed by the Faculty of Medicine, and enacted by the Senate.

1. No one fhall be admitted to the degree of Doctor of Medicine, except on the 24th of June and the 12th of September, or the days immediately fubfequent.

2. No one fhall be admitted into the number of candidates unlefs he fhall have, for the fpace of three years, ftudied, in this or fome other Univerfity, Medicine and the branches comprized in it, to wit, *Anatomy and Surgery*, *Chemiftry*, *Botany*, *Materia Medica and Pharmacy*, and the *Theory and Practice of Medicine*, and unlefs he fhall have attended the Clinical Lectures, given by the Profeffors of Medicine, on the cafes in the Infirmary.

3. Three months at leaft before the above-mentioned days, every one defirous of the honours of Medicine, muft deliver a *Medical Inaugural Dissertation* to one of the Medical Profeffors, who is to read it over, amend it if neceffary, and annex to it, in writing, his *perlegi*, or certificate of having read it, with the day on which it was received.

4. On

4. On or before the 20th of April, or July, he muſt communicate his wiſh to the Dean of the Faculty of Medicine;* and at the ſame deliver to him, his Inaugural Diſſertation (with the certificate of the Profeſſor who has read it annexed) to be ſubjected to the opinion of the Faculty of Medicine, in order that they may, by this ſpecimen, as well as by a private examination, be acquainted with his progreſs in learning, particularly in the ſcience of medicine, before they admit him into the number of Candidates.

5. On the 18th of May, or the 6th of Auguſt, the Candidate ſhall be examined by two medical Profeſſors before the Faculty of Medicine, in the various branches of medical ſcience above enumerated.

6. Should the Candidate be approved, an aphoriſm of Hippocrates ſhall be propoſed to him by one of the medical Profeſſors, and a medical queſtion by another. The former, explained and illuſtrated by a commentary; the latter, with an anſwer ſupported by proper arguments, he ſhall deliver on the 28th of May, or the 16th of Auguſt, to the Profeſſors who propoſed them; and he ſhall defend his commentary and anſwer, on the 30th of May, or the 18th of Auguſt, before the Faculty of Medicine.

7. Should the Candidate be thus far approved, there ſhall be delivered to him by two Profeſſors, two hiſtories of diſeaſes, with queſtions annexed, that he may illuſtrate and anſwer them in writing. The caſes thus illuſtrated, together with the anſwers, he ſhall deliver to the Profeſſors propoſing them on the 12th of June or the 1ſt of September, and ſhall defend them before the Faculty of Medicine on the 15th of June or the 3d of September.

8. If the candidate be approved after the firſt trial on the 18th of May, or the 6th of Auguſt, he is at liberty to print his Inaugural Diſſertation, and muſt deliver ſix correct copies of it (wanting the page which expreſſes the title and the authority of the Univerſity) to the Dean of the Faculty of Medicine, on the 15th of June, or the 3d of September.

9. Should the Candidate be a third time approved by the Faculty of Medicine, the Dean of the Faculty ſhall report to the Senate every thing which has been done, and by their authority and approbation, he ſhall be ordered to publiſh and defend it, on the 24th of June or the 12th of September. He ſhall then, if agreeable to the Senate, receive in the uſual way, the

* This is generally, if not always, the youngeſt Profeſſor of that Faculty.

the reward of his ſtudies and labour, the degree of Doctor of Medicine.

10. The Faculty of Medicine ſhall always meet at the College on the above mentioned days at nine o'clock in the morning.

11. All the aforeſaid examinations and exerciſes ſhall be in the Latin language.

No examinations can be conducted with more candour than thoſe in Edinburgh. Queſtions are never aſked with a view to puzzle or perplex, nor are any ever propoſed which a ſtudent ſhould not be able to anſwer. On the other hand, the examinations are not merely formal, as the Profeſſors never heſitate in rejecting a man, whom they do not conceive properly qualified. The principal examination is that mentioned as a private one at No. 4 of the Statuta Solemnia; it is held at the houſe of one of the Profeſſors, and is attended by the whole of the Faculty of Medicine as examinators. The gentleman at whoſe houſe the meeting is held, begins by an examination of the general functions of the body, and then ſelects a particular viſcus, of the ſtructure and uſes of which he requires an explanation. The others confine themſelves to practical and pathological queſtions on the diſeaſes of this organ, in which the prognoſis, diagnoſis, treatment, and nature of the remedies employed in the cure, come under review.

The other examinations are in the College Hall. The degree is conferred by the Principal, after an oath taken by each Graduate to practice his profeſſion in a juſt and honourable way. The diplomas are ſigned by the Principal and the whole of the members of the Senatus Academicus.

The graduation fees to the College amount to between thirteen and fourteen pounds, and are paid to the proper officer immediately after the examination at No. 5 of the ſtatutes. A gentleman who has paſſed this examination may, if he pleaſe, defer taking his degree for ſome time, and reſume his exerciſes at the ſtage at which they were left off.

Having given you this general account of the mode in which medical education is conducted in the College of Edinburgh, I ſhall take an early opportunity of tranſmitting you ſome miſcellaneous particulars and obſervations on the ſubject.

*London, September 8, 1801.* Y.

